# A highly sensitive trap vector system for isolating reporter cells and identification of responsive genes

**DOI:** 10.1093/biomethods/bpy003

**Published:** 2018-04-24

**Authors:** Kosuke Ishikawa, Yuta Kobayashi, Yutaro Wakabayashi, Shinya Watanabe, Kentaro Semba

**Affiliations:** 1 Japan Biological Informatics Consortium (JBiC), 2-45 Aomi, Koto-ku, Tokyo 135-8073, Japan; 2 Department of Life Science and Medical Bioscience, School of Advanced Science and Engineering, Waseda University, 2-2 Wakamatsu-cho, Shinjuku-ku, Tokyo 162-8480, Japan; 3 Translational Research Center, Fukushima Medical University, 1 Hikarigaoka, Fukushima 960-1295, Japan

**Keywords:** trap vector, reporter cell, gene mining, transposon

## Abstract

We devised a versatile vector system for efficient isolation of reporter cells responding to a certain condition of interest. This system combines nontoxic GAL4-UAS and *piggyBac* transposon systems, allowing application to mammalian cells and improved expression of a fluorescent reporter protein for cell sorting. Case studies under conditions of *c-MYC* gene induction or endoplasmic reticulum (ER) stress with thapsigargin on mouse or human cell lines confirmed easy and efficient isolation of responsive reporter cells. Sequence analyses of the integrated loci of the thapsigargin-responsive clones identified responsive genes including *BiP* and *OSBPL9*. *OSBPL9* is a novel ER stress-responsive gene and we confirmed that endogenous mRNA expression of *OSBPL9* is upregulated by thapsigargin, and is repressed by IRE1α inhibitors, 4μ8C and toyocamycin, but not significantly by a PERK inhibitor, GSK2656157. These results demonstrate that this approach can be used to discover novel genes regulated by any stimuli without the need for microarray analysis, and that it can concomitantly produce reporter cells without identification of stimuli-responsive promoter/enhancer elements. Therefore, this system has a variety of benefits for basic and clinical research.

## Introduction

Reporter cells are among the most important tools to promote the development of research [1]. They can be utilized for many valuable purposes, such as drug or toxicity evaluation [2–4], connecting signal pathways [5, 6], and the selection of cells that survive a specific condition [7]. Among many alternatives, gene trap-based technology is the most powerful method to obtain reporter cells accompanied by responsive gene identification [8]. This technology uses a vector, which integrates randomly into a genome and is designed to express a reporter gene driven by the near *cis*-acting promoter/enhancer elements.

By taking advantage of this, it is possible to conduct broad genome-wide screening without using a microarray and to identify even a minor change of gene expression buried in a sea of other genes. It also allows direct usage of isolated clones as reporter cells without the difficulty of determining the promoter/enhancer region.

A green fluorescent protein (GFP)-reporter-based retroviral gene trap vector system was previously developed [9]. This system works well but could be improved upon, mainly because of a low level of fluorescent reporter gene expression making it difficult, particularly in transient conditions, to segregate stimulus-dependent clones from the enormous population of other negative cells. In addition, the efficiency of viral vector packaging would be lower when an introduced trap cassette sequence is made longer or more complicated, such as for transcription termination, to improve its sensitivity. It is also known that retroviral genomic integration preferentially occurs upstream of actively transcribed genes [10–12], restricting the number of gene targets. To resolve these problems, a GAL4-UAS system was adopted to improve the sensitivity of the trap vector. In addition, the *piggyBac* transposon vector system was employed as a delivery vehicle for rapid and easy transfection, creating almost no limitations regarding the delivered DNA elements and the length of the trapping cassette, and practically overcoming the limitation of there being a preferential site for genomic integration. Here, we report the dramatic improvement of the trap vector system, especially in its sensitivity, and case studies of the isolation of reporter cells responding to c-MYC gene induction and endoplasmic reticulum (ER) stress by thapsigargin.

## Material and methods

### Vector construction

All used vectors are listed in Supplementary Table S1. polymerase chain reaction (PCR), enzymatically digested, and/or annealed oligo DNA fragments were joined by either ligase reaction (Nippon Gene, Tokyo, Japan, or Takara, Kyoto, Japan) or recombinant reaction by In-Fusion (Takara).

### Cell culture, viral infection, and plasmid transfection

NMuMG cells (kindly provided by K. Miyazawa, University of Yamanashi, Japan) were cultured in Dulbecco's modified Eagle's medium (DMEM) supplemented with 10% fetal bovine serum (FBS), 100 µg/ml streptomycin sulfate, 100 U/ml penicillin G potassium (SMPG), 10 µg/ml insulin, and 0.45% glucose. HeLa cells were cultured in DMEM (10% FBS, SMPG). One day before transfection, 2 × 10^5^ cells/well for NMuMG cells or 7 × 10^4^ cells/well for HeLa cells were plated on 12-well plates. Transposon helper and donor vectors were introduced by pouring the mixture (the ratio is shown in Tables 1 and 2) of 1–2 µg of DNA and 4–6 µg of polyethylenimine (PEI) (Polysciences, Warrington, PA) into 100 µl of Opti-MEM for a culture volume of 1 ml. The procedure for the preparation of retroviral and lentiviral vectors and their infection was previously described [13]. Pantropic VSV-G was used as their envelope. The HeLa cells used for screening thapsigargin-responsive cells were yielded by three repeated treatments of 10 nM thapsigargin overnight and expansion. For analysis or screening, cells were treated overnight or for the time indicated with 100 nM thapsigargin (Sigma, St Louis, MO), 10 µg/ml tunicamycin (Sigma), or 200 µM forskolin (Wako). When IRE1α inhibitors [4μ8C (Sigma), and toyocamycin (Cayman Chemicals, Ann Arbor, MI)] or a PERK inhibitor (GSK2656157, Adooq Bioscience, Irvine, CA) were used, cells were treated with these reagents for 1.5–2 h before the addition of 100 nM thapsigargin and collected after a further 15 h or the time indicated.

**Table 1:** Summary of gene trap screening by *c-MYC* transient expression in NMuMG cells

Cell line	Vector type (refer to Fig. 1)	Donor vector: helper (transposase) vector (µg)^a^	Transfection number of 7 × 10^4^ cells/well seeded onto 12-well plate	Ave. EGFP(+)% before first negative sorting^a^	Positive colonies^b^	Independent responsive clone
NMuMG Tet3G-IRES-neo^R^, TRE3G-*c-MYC*-hmKeimaRed	IRES	1: 2	1	1.26	0	
NMuMG Tet3G-IRES-neo^R^, TRE3G-*c-MYC*-hmKeimaRed	FrameA	1: 2	2	0.70	14^c^	1 (***Hsph1***)
	FrameC	1: 2	2	0.57	0	
NMuMG Tet3G-IRES-hygro^R^, TRE3G-*c-MYC*-hmKeimaRed	FrameA	1: 1	4	0.75	0	
	FrameB	1: 1	4	0.47	0	
	FrameC	1: 1	4	0.67	0	
	FrameA	3: 1	4	0.72	2	1 (***Ddx21***)
	FrameB	3: 1	4	0.50	0	
	FrameC	3: 1	4	0.53	0	

^a^See discussion;

^b^number of clones singly sorted into a 96-well plate and showing EGFP(+) only under Dox addition;

^c^see Supplementary Figure S5 for splinkerette PCR analysis.

**Table 2:** Summary of gene trap screening in HeLa cells by drug stimuli

Cell line and drug	Vector type (refer to Fig. 1)	Donor vector: helper (transposase) vector (µg)^a^	Transfection number of 2 × 10^5^ cells/well seeded onto 12-well plate	Ave. EGFP(+)% before first negative sorting^b^	Positive colonies^c^	Independent responsive clones^d^
HeLaS3 + forskolin addition	IRES	1: 2	6	1.14	6	2
HeLaS3 thapsigargin^R^ + thapsigargin addition	FrameA	2: 1	4	0.58	13	3 (including ***OSBPL9***)
	FrameB	2: 1	3	0.36	3	2
	FrameC	2: 1	4	0.36	14	1 (***HSPA5***(***BiP***))

^a^See discussion;

^b^see discussion;

^c^number of clones singly sorted into a 96-well plate and showing EGFP(+) only under Dox addition;

^d^we inferred these from the band patterns of electrophoresis of splinkerette PCR products. Not all integration sites were determined by sequence.

### Fluorescence-activated cell sorting

Cells dissociated by 0.05% trypsin–0.5 mM ethylenediaminetetraacetic acide phosphate-buffered saline (EDTA–PBS) were resuspended in 2% FBS–Hanks' balanced salt solution (HBSS) and filtered through a 48-µm-pore mesh (Sanplatec, Osaka, Japan). Sorting was carried out using a cell sorter, SH800Z (Sony, Tokyo, Japan).

### Splinkerette PCR

We performed splinkerette PCR [14] using a slightly modified version of a previously described protocol [15]. Annealed adaptor DNA composed of 5′-CGAAGAGTAACCGTTGCTAGGAGAGACCGTGGCTGAATGAGACTGGTGTCGACACTAGTGG-3′ and 5′-GATCCCACTAGTGTCGACACCAGTCTCTAATTTTTTTTTTCAAAAAAA-3′ was ligated to BstYI (PsuI, Fast Digest, Thermo Fisher Scientific, Waltham, MA)-digested genomic DNA purified by NucleoSpin Tissue kit (Macherey-Nagel, Duren, Germany) using T4 DNA ligase (Nippon Gene). Then, nested PCR was performed in conditions shown in Supplementary Table S2. PCR fragments after agarose electrophoresis were recovered using Ultra Clean purification kit (MO BIO Laboratory, Carlsbad, CA) and sequenced by ABI3130 (Applied Biosystems, Foster City, CA).

### Real-time PCR

RNA was extracted from the cultured cells using Isogen (Nippon Gene). Reverse transcription was performed using the SuperScript III First-Strand Synthesis System (Thermo Fisher Scientific) with random hexamer as a primer mix. THUNDERBIRD SYBR qPCR mix (TOYOBO, Osaka, Japan) was used for real-time PCR, which was executed using the StepOnePlus Real Time PCR System (Applied Biosystems). Human *HPRT1* was used as a relative control. Primers are listed in Supplementary Table S3.

### Immunoblotting

Cells were pretreated with the indicated concentration of GSK2656157 for 1.5 h, followed by the addition of 100 nM thapsigargin for the indicated times. Cells were washed with PBS containing 50 mM NaF, 17.5 mM β-glycerophosphate, and 100 µM Na_3_VO_4_ and lysed with LysisB (50 mM Tris-HCl, pH 7.5, 150 mM NaCl, 1% NP-40, 0.1% sodium dodecyl sulfate (SDS), 100 mM NaF, 17.5 mM β-glycerophosphate, 100 µM Na_3_VO_4_, 10% glycerol). Cleared lysates were mixed with Laemmli sample buffer containing 0.6 M β-mercaptoethanol, followed by boiling, SDS–PAGE, and immunoblotting. Antibodies for immunoblotting were as follows: anti-CREB-2 (ATF4, C-20, Santa Cruz, Dallas, TX), anti-c-Myc (9E10, Santa Cruz), and anti-α-tubulin (Wako, Osaka, Japan).

### Luciferase assay

After washing once with PBS, cells were lysed with LCβ buffer (Toyo Inki, Tokyo, Japan) at room temperature for over 30 min with intense agitation. Luciferase activity in the cleared lysates was measured by mixing a luciferin substrate (Promega, Madison, WI) using TriStar^2^S LB942 (Berthold Technologies, Bad Wildbad, Germany). The values of luciferase activity were standardized by the protein concentration titered by the BCA protein assay kit (Thermo Fisher Scientific).

## Results

### Construction of highly sensitive trap vector

**Figure 1:**
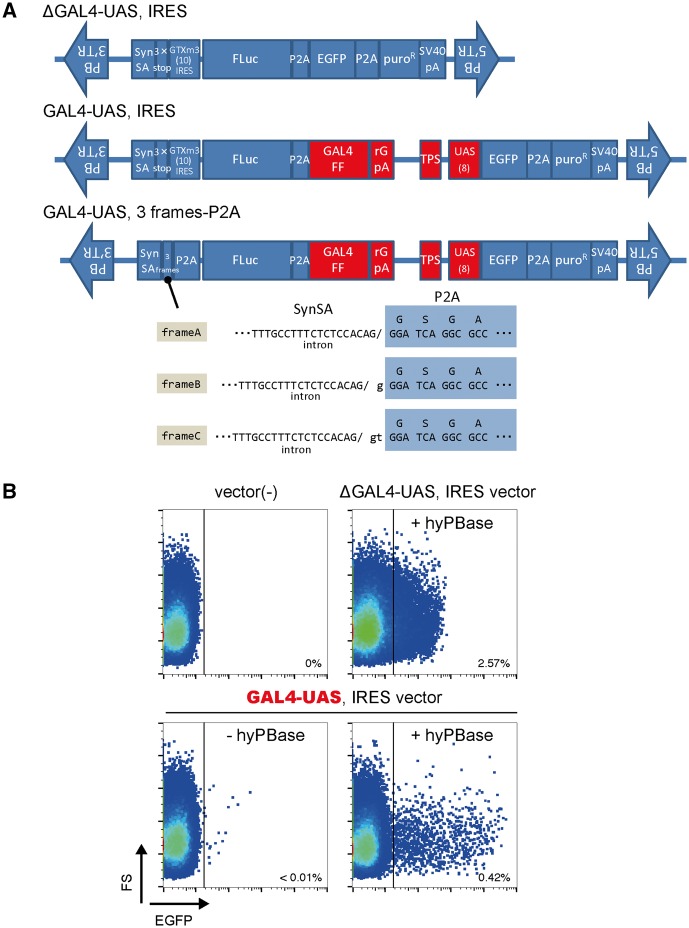
Establishment of highly sensitive trapping vectors dramatically improving reporter expression. (**A**) Structures of old (ΔGAL4-UAS) and developed (GAL4-UAS, shown in red) transposon donor vectors. Syn SA, synthetic splicing acceptor; rGpA, rabbit globin polyadenylation signal; TPS, transcription pause site. Numbers in parentheses show the repeat number. (**B**) Evaluation of EGFP reporter expression. HeLa cells were cotransfected with the old or new IRES vector with or without the transposase (hyPBase) helper vector. After 11 days, cells were collected and analyzed using a flow cytometer.

To increase the sensitivity of promoter/enhancer trap vectors, we utilized a nontoxic GAL4-UAS system developed for vertebrate species [16, 17]. The modified Gal4, called GAL4FF, consists of an extremely trimmed minimal DNA-binding site of yeast Gal4 transcription factor and a few repeats of minimal transcription activation module from VP16. This engineered Gal4 can bind to an upstream activating sequence (UAS) and strongly activates downstream reporter genes. We placed eight repeats of 5′-cggagtactgtcctccgag-3′ UAS upstream of the *EGFP* gene (Fig. 1A).

For a vehicle for delivering a trapping cassette, we chose a transposon vector system [18], which allows fairly random integration events compared with viral vector systems [11, 19], loading of a long DNA element (over 100 kb) [20–22], as well as rapid and easy manipulation upon introduction into cells. We inserted trapping modules between the TR (terminal repeat)/IR (inverted repeat) sequences of *piggyBac* transposon in the 3′-to-5′ direction of its original designation to reduce leaky expression activity [23]. By coexpression of helper transposase vector [here, we use the hyperactive variant of *piggyBac* transposase (hyPBase) [24]], DNA elements between TR/IRs can be integrated into the host genome via a cut-and-paste mechanism. At the head of the trapping module, a strong synthetic splicing branch and acceptor sites were placed, aiming to receive the splicing donor of an endogenously expressed transcript. To express GAL4FF at trapped loci, we designed two types of vector structure. One is a three-frame stop codon followed by IRES (10 repeats of Gtx-m3 [25, 26], Fig. 1A, GAL4-UAS, IRES). The other has three-frame patterns of *porcine teschovirus-1*’s 2 A self-cleaving peptide configuration [27–29] (Fig. 1A, GAL4-UAS, three-frame P2A). In our observation, the latter (three different frame vectors) is more stable (Supplementary Figure S1) and has lower, although occasionally appropriate, trapping efficiency. Besides the EGFP reporter gene, a firefly luciferase (Fluc) gene is introduced with the aim of proceeding to a quantitative assay after the isolation of reporter cells. Here, we introduced the Luc2CP gene (from pGL4.16; Promega) encoding an unstable version of Fluc by hCL1 and hPEST destabilization peptide sequences in front of GALFF, linked by a P2A sequence.

To test whether the GAL4-UAS trapping vectors work in human cells, we cotransfected the helper and donor vectors into HeLa cells. Compared with non-GAL4-UAS vector (Fig. 1A, ΔGAL4-UAS, IRES), our developed vector dramatically enhanced the expression of EGFP reporter protein (about 100-fold of max. EGFP signal) to ensure easy differentiation from EGFP-negative cells (Fig. 1B). Importantly, the EGFP expression levels were evenly distributed from low to high, suggesting that the donor sequence is inserted into myriad loci without a strong bias of gene expression and of transcriptional amplification by the GAL4-UAS system.

### Case studies using the highly sensitive trap vector system for isolating reporter cells

#### Isolation of mouse reporter cells responsive to c-MYC gene expression

**Figure 2:**
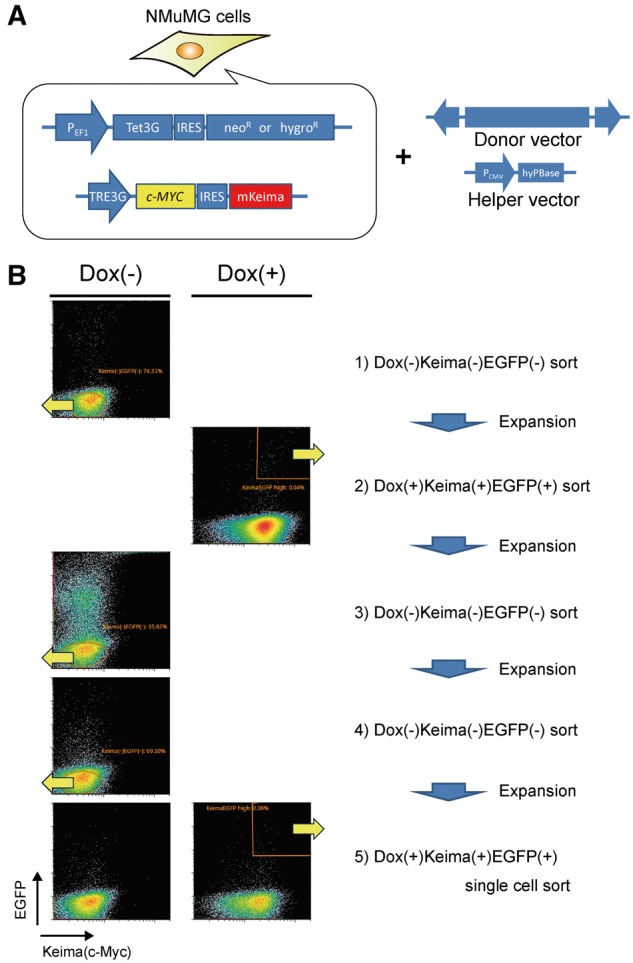
Cloning of *c-MYC*-responsive cells. (**A**) Structure of vector system in NMuMG cells for repeated induction of *c-MYC* expression. After the cells, in which *c-MYC* is strongly expressed by doxycycline administration, had been established (Supplementary Figure S2), one of the trapping vectors (Fig. 1A) was co-introduced with a helper (transposase) vector (here, we used hyPBase [24]). (**B**) Procedure for isolation of cells responsive to *c-MYC* expression. The cells transfected with the trapping vectors were treated without (−) or with (+) 100 ng/ml doxycycline (Dox) for 1 day. Cells were collected and fluorescence-activated cell sorting was performed. The collected area is indicated by yellow arrows. In some cases, steps (3) and (4) were conducted repeatedly until decreases in the proportion of stably expressing EGFP(+) cells, which were unwanted, no longer occurred.

**Figure 3:**
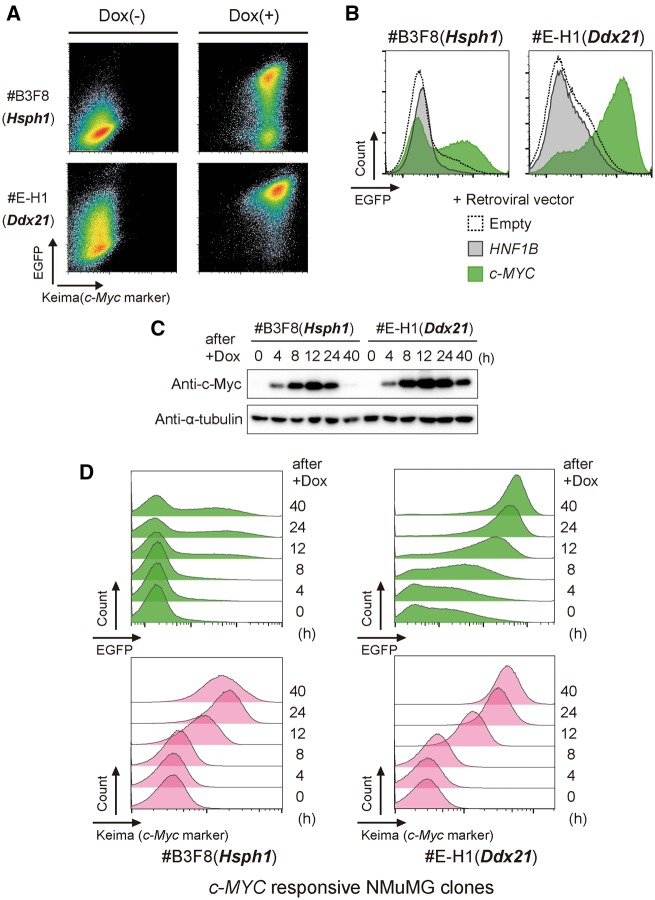
(**A**) Flow cytometric analysis of successfully isolated clones. Clones isolated from the *c-MYC*-responsive NMuMG cells shown in Fig. 2B, were treated without (−) or with (+) 100 ng/ml doxycycline and, after 38 h, flow cytometric analysis was performed. (**B**) Confirmation of reporter expression in response to *c-MYC* expression. The isolated *c-MYC*-responsive clones induced EGFP reporter expression by infection with retroviral expression vectors for *c-MYC*, indicating that the reporter expression in the above clones was really caused by *c-MYC* expression, not by a response to doxycycline. *HNF1B*, a gene unrelated to *c-MYC*, did not induce EGFP reporter, also demonstrating the specificity of the response to *c-MYC*. (**C**) Immunoblot analysis of c-Myc. The *c-MYC*-responsive cells were treated once with 100 ng/ml doxycycline and after the time indicated cells were collected and lysed, followed by SDS–PAGE and immunoblotting. (**D**) Time course analysis of the *c-MYC*-responsive cells using flowcytometer. Lower panels show expression of Keima, used as c-Myc expression marker (Fig. 2A).

To demonstrate the practicality of our system, we first attempted to isolate cells responding to the expression of a gene of interest. Here, we tested a transcription factor, *c-MYC*. *c-MYC* is one of the most well-known oncogenes, which, in most known typical mechanisms, functions in heterodimeric form to activate many genes involved in cell cycle progression and survival. We chose the mouse mammary epithelial cell line, NMuMG, because it is known to be regulated by *c-MYC* expression in tumor aggressiveness [30] and can expand from a single cell, which is essential for the cloning step. To be able to switch the gene expression on and off repeatedly, which is important for extracting highly responsive cells, we introduced the Tet-On system into the NMuMG cells, where *c-MYC* expression is monitored by Keima (hmKeimaRed) [P_*EF1*_-Tet3G-IRES-neo^R^ or hygro^R^, TRE3G-*c-MYC*-IRES-Keima, Fig. 2A). To obtain cells that can strongly induce *c-MYC en masse*, we performed several cycles of positive and negative cell sorting under doxycycline (Dox(+) and Dox(−) conditions, respectively (Supplementary Figure S2)]. After the strongly *c-MYC*-inducible cells had been established, the cells seeded at approximately 7 × 10^4^ per sample for transfection were co-transfected with the donor (Fig. 1A) and hyPBase helper vectors. After another repeated session of negative and positive sorting under Dox(−) and Dox(+) conditions, respectively, we obtained two clones from a total of about 2 × 10^6^ seeded cells for transfection (Figs 2B and 3A, Table 1). Specific reaction upon *c-MYC* expression was confirmed by a retroviral expression system other than the Tet-On system (Fig. 3B). Doxycycline-induced c-Myc protein expression was also confirmed by immunoblot analysis (Fig. 3C). The expression level of induced c-Myc protein was declined at 24 h after induction probably by degradation mechanism or doxycycline inactivation. Time course analyses of these clones after doxycycline induction by flowcytometer revealed that the time lag between EGFP expression and Keima expression was hardly recognized (Fig. 3D). Despite single cell sorting for the clone isolation, #B3F8 clone apparently had nonresponsive cell population even at 24 h after Dox induction. In contrast, #E-H1 clone showed responsive in almost all cells. Note that in our drug-responsive reporter cell collections, we observed some clones showed decreased reactivity after multiple passage, which was not recovered even after a further cloning procedure, while other clones inversely increased reactivity after enrichment of a responsive fraction or after a further cloning procedure. The mechanism of the determinants of this cell fate decision is still unclear, but one possibility is a reversible or irreversible feedback mechanism by epigenetic regulations such as DNA methylation. Genomic mapping of the integration sites by splinkerette PCR [14] and subsequent sequence analyses identified two known c-Myc-regulated genes (Supplementary Figure S3). One is *Hsph1* (heat shock protein H1; also known as *Hsp105*), known to be expressed by c-Myc in human leukemia cells (e.g. see ref. [31]), which was recently demonstrated to physically bind to c-Myc protein as a chaperone and is required for aggressiveness in human lymphoma [32]. Another is *Ddx21* [DEAD (Asp-Glu-Ala-Asp) box helicase 21], which has been demonstrated to be a coexpression marker with *c-MYC* in colorectal cancer [33] and is known to be directly transcribed by c-Myc [34]. These results demonstrate that our system easily isolates responsive clones and has great potential for identifying downstream or target genes by chance.

#### Isolation of HeLa reporter cells responsive to external stimuli

**Figure 4:**
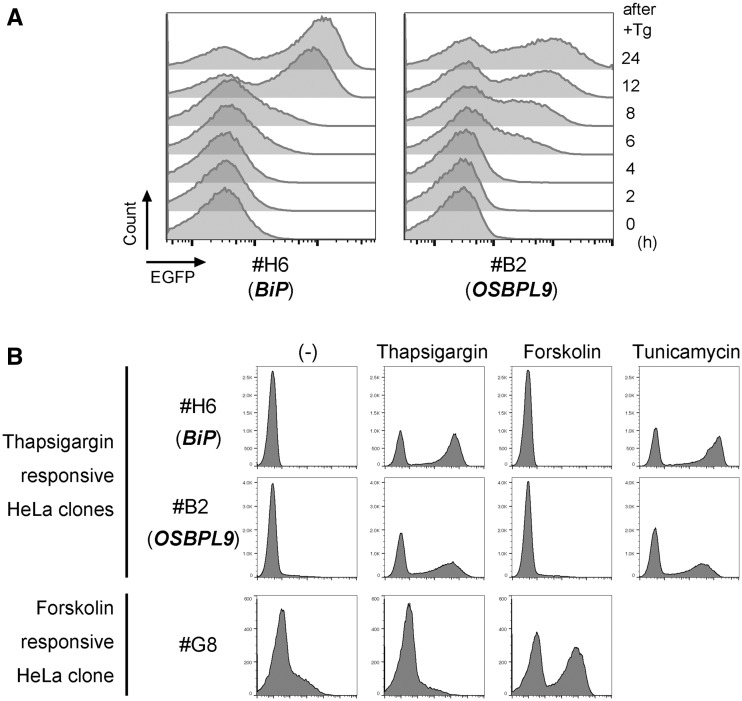
Cloning of thapsigargin-responsive cells. (**A**) Time course analysis of reporter expression of isolated clones responsive to thapsigargin (Tg). Clones were treated with 100 nM thapsigargin. After the time indicated, flow cytometric analysis was performed. (**B**) Specific responsivity of HeLa clones isolated under thapsigargin or forskolin stimulus. Forskolin responsive clone was similarly isolated (Table 2) and used as a control. Thapsigargin-responsive clone specifically reacted with thapsigargin, but not with forskolin. Thapsigargin-reacted clones also responded to the glycosylation inhibitor tunicamycin, both of which are known to induce ER stress.

**Figure 5:**
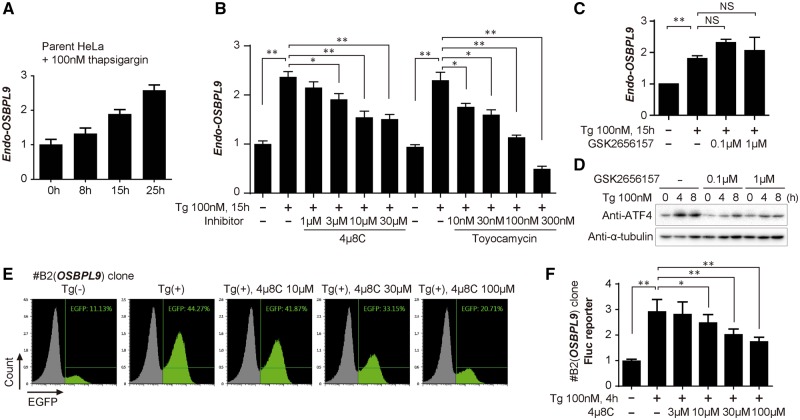
Evaluation of thapsigargin response of the *OSBPL9* gene. (**A**) RT–PCR analysis of induced expression of endogenous *OSBPL9* mRNA by Tg treatment. A representative result of similar results from two experiments is shown. *F* = 81.35 and *P* < 0.0001 by one-way ANOVA of 3–6 repeated data measurements of the representative experiment. (**B**) RT–PCR analysis for the effects of IRE1α inhibitors 4μ8C and toyocamycin on Tg-induced *OSBPL9* expression. A representative result of similar results from two experiments is shown. *P < 0.001, **P < 0.0001 by *t*-test of four repeated data measurements of the representative experiment. (**C**) RT–PCR analysis for the effects of the PERK inhibitor GSK2656157 (1.5 h) on Tg-induced *OSBPL9* expression. *n* = 3, **P < 0.0001 by *t*-test, NS, not significant. (**D**) Immunoblot analysis using anti-ATF4 for confirming the repression of the PERK pathway by GSK2656157 shown in (C). Anti-α-tubulin blot is shown as a loading control. (**E**) Evaluation of thapsigargin response with EGFP reporter expression. #B2 clone, in which the *OSBPL9* gene was trapped (Fig. 4A), was treated with the indicated concentration of 4μ8C for 2 h, followed by the addition of 100 nM thapsigargin for 15 h and analysis by a flow cytometer. (**F**) Evaluation of thapsigargin response with Fluc reporter expression. #B2 clone was treated with the indicated concentration of 4μ8C for 2 h, followed by the addition of 100 nM thapsigargin for 4 h. Cell lysates were subjected to Luc assay. *n* = 9 in three experiments, *P < 0.05, **P < 0.0001 by *t*-test.

We next investigated whether this system could also isolate a cell clone responding to certain external stimuli. For this purpose, we tested two arbitrarily chosen reagents administered to HeLa cells. One is thapsigargin, best known for its activity of inhibiting Ca^2+^ ion pump ATPases residing in intracellular membranes. The other is forskolin, known for activating adenylate cyclase. The introduction of donor and helper vectors into HeLa cells, followed by a few cycles of negative and positive cell sorting under stimulus (−) and (+), respectively, resulted in the successful isolation of two independent clones for forskolin and six independent clones for thapsigargin, from totals of 1.2 × 10^6^ and 2.2 × 10^6^ seeded cells, respectively (Table 2). Sequence analyses of its integration sites (Supplementary Figure S4) revealed that one of the corresponding genes for thapsigargin is *HSPA5* (also known as *BiP* or *GRP78*) [35] and another is a novel gene for responding to thapsigargin, *OSBPL9* (also known as *ORP9*), known for regulating Golgi structure and function through cholesterol binding and transfer activity [36–38]. Time course analysis revealed that these clones expressed EGFP reporter protein detected only 6 h after administration (Fig. 4A). These clones did not react with another unrelated reagent (Fig. 4B), confirming the specificity of responsiveness to each stimulus. In contrast, the thapsigargin-responsive clones responded to the glycosylation inhibitor tunicamycin as they had responded to thapsigargin, both of which are known to induce ER stress (Fig. 4B, right panels). We confirmed that endogenous *OSBPL9* mRNA is induced by thapsigargin (Fig. 5A). To determine which pathway is involved in *OSBPL9* expression upon ER stress, several known inhibitors were tested. *OSBPL9* mRNA was repressed by the IRE1α inhibitors 4μ8C and toyocamycin (Fig. 5B), but not significantly by the PERK inhibitor GSK2656157 (Fig. 5C and D), suggesting that *OSBPL9* is regulated by the IRE1 pathway upon the unfolded protein response [39]. The EGFP reporter expression and the firefly luciferase reporter expression (Fig. 1A) by thapsigargin in the isolated cell clone (#B2) were also inhibited by 4μ8C (Fig. 5E and F), similar to the endogenous gene expression (Fig. 5B). These results demonstrate that this trapping system enables us to isolate reporter cells accompanied by the identification of novel regulated genes.

## Discussion

### Improvement of the reporter gene expression of the trap vector

Isolating reporter cells by using trapping technologies has an advantage over producing them by a knock-in or transgenic method. Specifically, it does not require consideration of responsive genomic elements and their length as well as the distance from a reporter gene to which they are linked. For reporter cell isolation, both drug-resistant selection and cell sorting approaches would be considered. The drug-resistant approach requires prolonged culture during positive cell selection to remove negative cells, and thus is restricted to limited conditions leading to a long-lasting response of cells. In contrast, a cell sorting approach can be used more broadly, even in transient stimulus conditions, if its expression is sufficient to detect. Moreover, both weakly and strongly responsive cells can be selected directly. Genes encoding fluorescent proteins have been used as reporter genes in the cell sorting approach, but their expression levels in conventional systems using EGFP were too low to segregate positive cells from the enormous number of negative ones, especially in transient stimulus conditions. In this study, we demonstrated that the amplification of reporter gene expression by the GAL4-UAS system dramatically improved trapping sensitivity (Fig. 1B), solving this problem. Using EGFP as a reporter, the newly devised system can detect responsive signals as early as 6 h after stimulus (Fig. 4A).

This ability enables us to isolate reporter cells efficiently. In fact, our improved trap vectors identified the *OSBPL9* gene as a gene that responds weakly but significantly to ER stresses. This gene was not found among the top 250 significantly regulated candidate genes (as determined by GEO2R analysis) among public microarray data available from the NCBI Gene Expression Omnibus (GEO) for samples of human cell lines treated with thapsigargin. Among the public microarray data, we found that, in human neuroblastoma cells, SH-SY5Y (GEO accession: GSE24497) and IMR-32 (GSE6976) [40], *OSBPL9* expression is significantly upregulated upon thapsigargin treatment. However, its ranking of statistical significance (by *P*-value) for the difference in expression was too low to identify *OSBPL9* as an ER-stress-responsive gene. This example illustrates the difficulty in identifying a regulated gene from many catalogs of microarray data. Genetic screening as performed in this study is thus considered to be a more attractive method to highlight novel regulated genes.

### Technical considerations for efficient isolation of reporter cells and determination of responsive genes

To optimize the efficiency with which reporter cells can be isolated, an optimal repertoire of trapped cells needs to be prepared. Through analyzing integration sites of the isolated clones from the same transfection sample, we noted that, occasionally, several unique integration sites other than one common site (e.g. *Hsph1* locus) were seen among probable sister clones. [In the *Hsph1* clone, we identified at least seven probable sisters out of 14 isolated colonies, which share a common integration site in the *Hsph1* gene (Supplementary Figure S5).] This suggests that, after the first genomic integration in the parent clone, transpositional events had continued during several cell divisions, presumably because of the presence of residual donor vector and transposase. If this is true, repertoire cell number could be unexpectedly but conveniently increased. This may be one reason why we can successfully isolate expected clones efficiently from a small-scale culture. Paradoxically, the large repertoire is not always desirable for reporter isolation. We occasionally observed that a small number of cells that express EGFP reporter could not be removed despite performing multiple cycles of negative sorting for removal of the population constitutively expressing EGFP reporter. One possible explanation for this is that a specific cell-cycle-dependent gene is trapped and cannot be removed from the bulk culture. This background could be a major obstacle to isolation of the target clone. Thus, for HeLa and NMuMG cells, we currently operate transfection using a 12-well plate [in which 7 × 10^4^ (NMuMG) or 2 × 10^5^ (HeLa) cells/well were seeded], preparing multiple lots, and discarding an undesired lot (about 1 out of 3–10 lots) containing a population of permanently unremoved cells expressing EGFP, and finally mixing desired lots (about 10–12) to obtain large repertoire. This kind of procedure may also be required to obtain responsive clones in other cell types.

Determination of the number of trap vectors integrated per cell is also important because the more excessively the trap vectors are integrated into a cell, the more frequently the expression specific to a certain condition is canceled out by other types of expression independent of the stimulus condition (such as constitutive expression). Moreover, the identification of the integration site, if necessary, becomes more difficult. We referred to the constitutively expressing EGFP(+)% of cells before the first negative sorting as an indicator for avoiding excessive multiple integrations and yet preserving a sufficient repertoire. This is a convenient approach that does not require Southern blotting or NGS analysis. The donor vector: helper vector ratio may be one of the most important conditions critically affecting the number of integrations per cell. In our experiments, if the donor vector ratio increased [up to 10: 1(OD_260_)], the EGFP(+)% and thus the integration number increased. Adjusting transfection time or total DNA amount also controlled the value of the EGFP(+)%. To avoid excessive integrations, we prepared transfected cells with a proportion of roughly 0.50%–2.0% EGFP(+)% when using PEI as a transfection reagent. Similarly, for other cell types or other transfection reagents, simultaneous preparation of transfected cells under several conditions and determination of the rough optimum range of EGFP(+)% may help to successfully isolate reporter cells.

Multiple integrations make it difficult to determine responsive genes. Fortunately, here we succeeded in identifying a gene (*OSBPL9*) as a novel candidate for responding to thapsigargin by splinkerette PCR and confirmed the responsive endogenous gene expression by RT–PCR. However, other responsive genes remain elusive in some clones (Table 2) due to the multiple integrations, and also due to the limited application by splinkerette PCR, which uses restriction enzyme digestion. Recent reports described an alternative method, named semiquantitative insertion site sequencing (QIseq), allowing high-throughput analysis for transposon insertion sites using acoustic shearing of DNA and optimized NGS [41–43]. These strategies based on genomic DNA amplification, however, are not appropriate to identify novel or poorly annotated transcripts. Therefore, 5′RACE or RNA-seq analyses would be an alternative approach because they can directly identify the trapped gene even if it expresses unannotated novel transcripts. These kinds of protocol should facilitate the precise identification of responsive genes in isolated reporter cells. Even after identification of a candidate responsive gene, there still remains the possibility that another trapped gene may have additional effect on the reporter gene expression. To rule out this possibility, specific removal of trap vector from the candidate gene by a genome editing technique may help to confirm that the identified gene is the sole factor responsible for the reporter expression.

In this study, we used only the *piggyBac* transposon system as a delivery vehicle for the trapping cassette. Other transposon systems (such as *Sleeping Beauty* [44] and *Tol2* [45]) with different properties are also available [46]. First, their target short recognition sequences differ (*piggyBac* targets TATA [47, 48], while *Sleeping Beauty* targets TA [49] and *Tol2* targets weak consensus sequences including AT-rich palindrome-like sequences [50]). In addition, integration preferences are known to differ among the systems. A report demonstrated that *PiggyBac* prefers to integrate into transcription start sites, while *Sleeping Beauty* displays more random integration [51]. Another report found difference in insertion preference among *PiggyBac*, *Sleeping Beauty*, and *Tol2* in analyses based on various factors involved in the 3 D organization of chromatin [52]. Therefore, their parallel use may provide further opportunities to find nonredundant-responsive elements [53].

### Vector variations for quantitative analysis and putative applications

Here, we used vectors with a degradative version of firefly luciferase gene for a quantitative assay. During the preparation of this manuscript, we also constructed additional vectors in which, e.g., a nondegradative type of Fluc gene is placed after UAS repeats to achieve high luciferase activities by accumulation. The availability of multiple vectors helps to optimize the evaluation system according to the purpose of the particular research for which it is used.

Basically, using this approach, it will be possible to isolate cells responding to a variety of other conditions, such as drug stimulation, tumor malignancy, and hypoxia. We believe that our developed tool is expected to be a powerful approach to directly and efficiently isolate reporter cells and identify responsive genes, which may be extremely useful for many basic and clinical applications.

## Availability

All vectors that we produced and predicted full sequences are available upon request.

## Supplementary data

Supplementary data are available at *Biology Methods and Protocols* online.

## Supplementary Material

Supplementary Data
