# Supplementary material for: A highly sensitive trap vector system for isolating reporter cells and identification of responsive genes
Source: Biol Methods Protoc. 2018 Apr 24;3(1):bpy003. doi: 10.1093/biomethods/bpy003 (PMC6994077; doi:10.1093/biomethods/bpy003)
Supplement: Supplementary Data [file bpy003_supp.pdf]

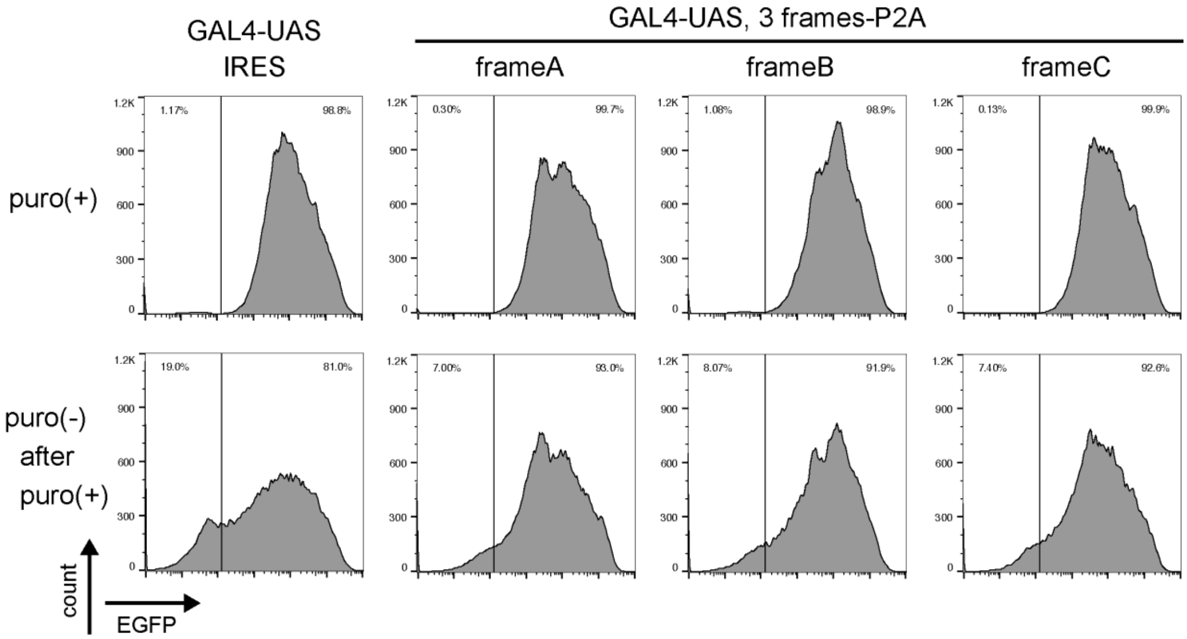

Figure S1 Stability of the reporter gene expression of the cells produced by the GAL4-UAS trap vectors depicted in Figure 1. The trap-vector-introduced NMuMG bulk cells (EGFP and puromycin resistance gene are to be expressed as reporters driven by constitutively activated promoters) were selected under 1 μg/mL puromycin for 7 days, followed by additional culture in the presence (upper panels) or absence of puromycin (lower panels) for 4 days. The cells were collected and analyzed using a flowcytometer. Although the mechanism involved is unknown, a small proportion of EGFP(−) cells appeared after puromycin selection had been released, which was more pronounced when Gtx-m3-IRES-type vector was used, compared with that for three-frame P2A-type vectors.

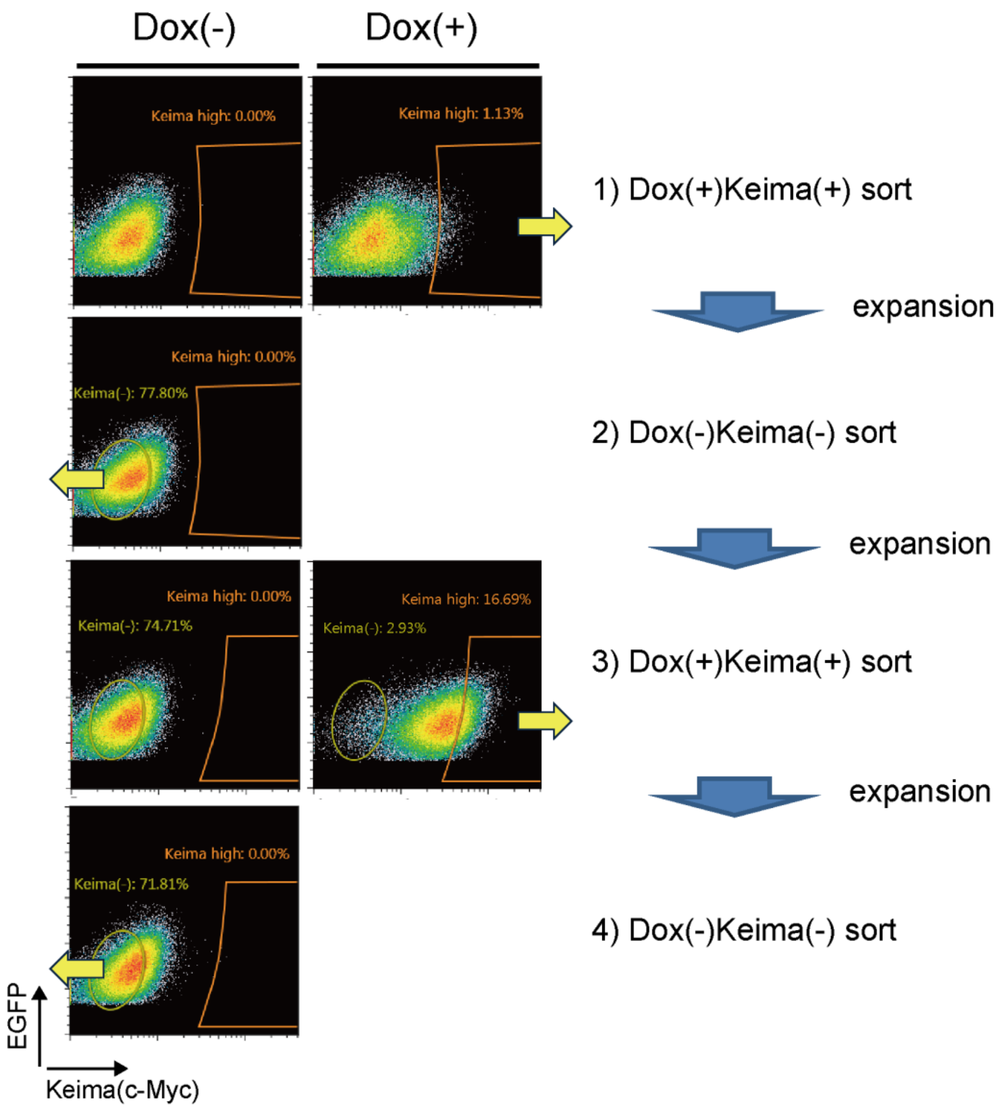

Figure S2 Procedure for producing reversibly *c-MYC*-inducible NMuMG cells. The cells transfected with the *c-MYC*-inducible viral Tet-On vector system shown in the left side of Figure 2A were sorted. Cells of Keima(+) (used as a red fluorescent marker for the expression of *c-MYC*) under Dox(+) or Keima(−) under Dox(−) were collected by FACS and subsequently expanded. The collected area is indicated by yellow arrows. We found that, for cells introduced with genes that cannot repeat this procedure, failure to isolate trapped cell clones frequently occurred.

#B3F8(***Hsph1***)

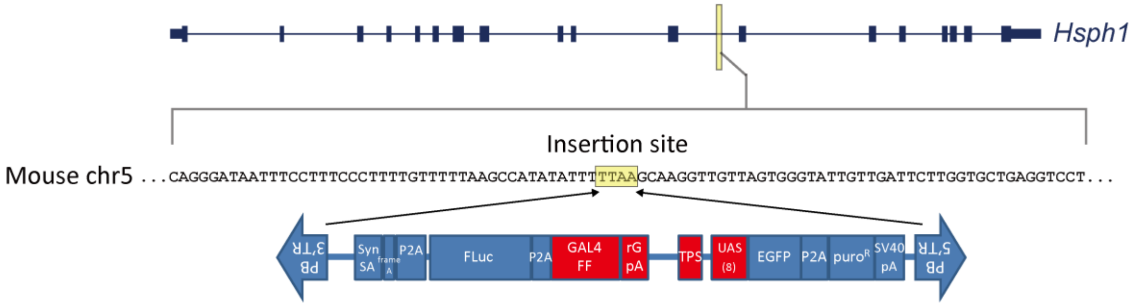

#E-H1(***Ddx21***)

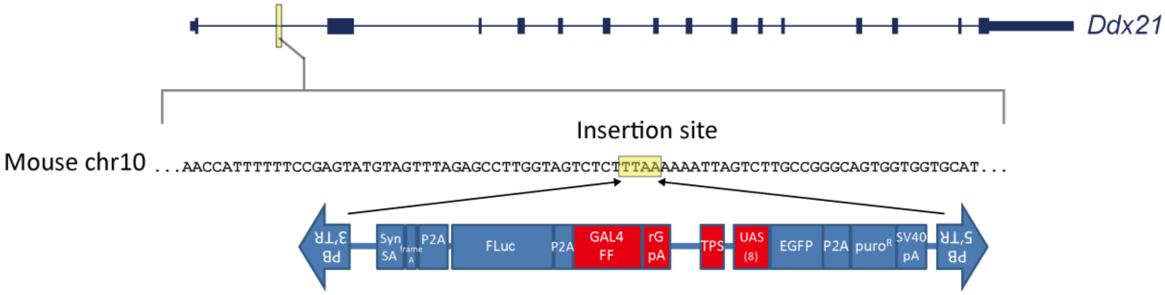

Figure S3 Genomic insertion sites of the trapping vector in the isolated *c-MYC*-responsive clones. Mapping was performed with UCSC Genome Browser using the sequence of the splinkerette PCR fragment.

#H6(***BiP***)

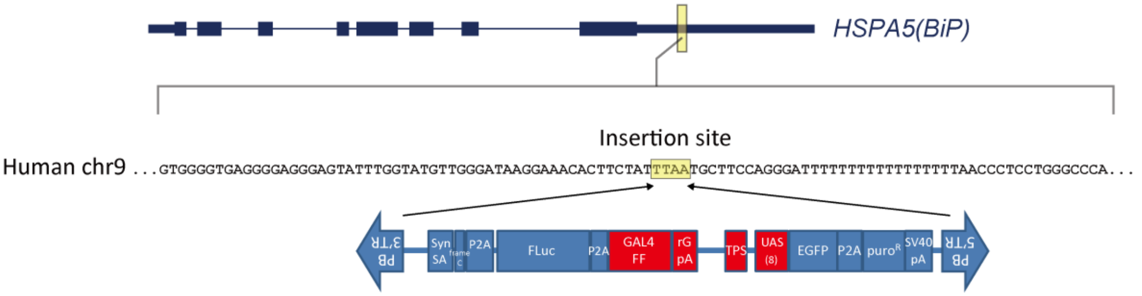

#B2(***OSBPL9***)

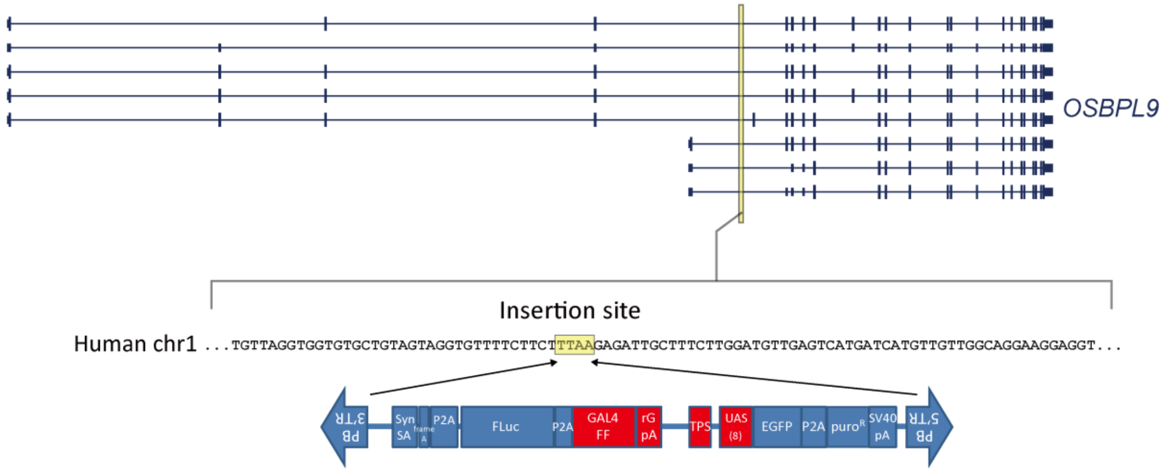

Figure S4 Genomic insertion sites of the trapping vector in the isolated thapsigargin-responsive clones. Mapping was performed with UCSC Genome Browser using the sequence of the splinkerette PCR fragment.

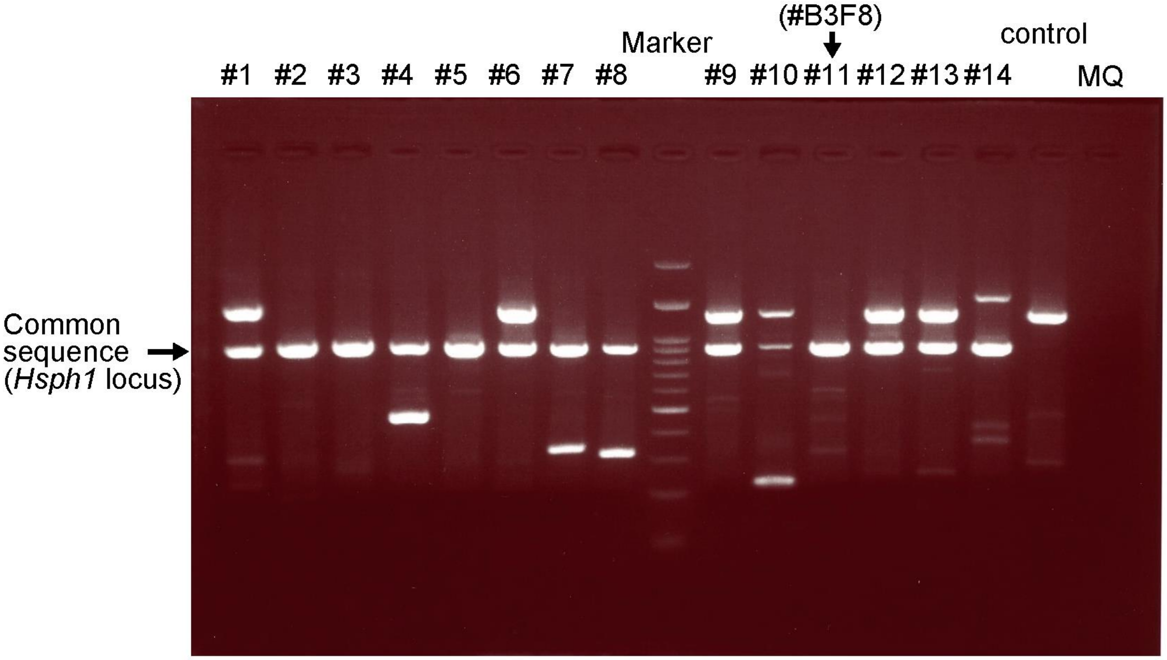

Figure S5 Image of agarose gel electrophoresis after splinkerette PCR. *c-MYC*-responsive clones from the same transfection sample (corresponding to *4 in Table 1) were subjected to splinkerette PCR to determine integration sites of the trapping vector. After the electrophoresis presented here, DNA fragments were recovered and sequenced. The sequencing results revealed that all clones had a common integration site in the *Hsph1* gene (Supplementary Figure S3). It was assumed that the common vector integration occurred first in a common ancestral cell, followed by the second hit [#1 (probably the same sister clone equivalent to #6, #9, #10, #12, #13), #4, #7, #8, and #14] after cell divisions presumably by residual transfected vectors. The third and additional hits might have occurred in #1, #10, and #14 (although this was not confirmed by sequencing). If these assumptions are true, the repertoire (having different integrated loci) can be increased from one cell. Control, a clone from another transfection sample. MQ, used for a PCR reaction control without a template.

Table S1. Vectors used in this study.

Vector name	Notes
pCMV-hyPBase	Kindly provided by the Wellcome Trust Sanger Institute (ref.(1))
pPB-SA-stop×3-GTXm3(10)IRES-FLuc-P2A-EGFP-P2A-Puro^{R}-SV40pA	Fig. 1A, ΔGAL4-UAS, IRES
pPB-SA-stop×3-GTXm3(10)IRES-<br>FLuc -P2A-GAL4FF-rGpA-TPS-UAS(8)-EGFP-P2A-Puro^{R}-SV40pA	Fig. 1A, S1 GAL4-UAS, IRES
pPB-SA-frameA-P2A-<br>FLuc-P2A-GAL4FF-rGpA-TPS-UAS(8)-EGFP-P2A-Puro^{R}-SV40pA	Fig. 1A, S1 GAL4-UAS, **frameA**-P2A
pPB-SA-frameB-P2A-<br>FLuc-P2A-GAL4FF-rGpA-TPS-UAS(8)-EGFP-P2A-Puro^{R}-SV40pA	Fig. 1A, S1 GAL4-UAS, **frameB**-P2A
pPB-SA-frameC-P2A-<br>FLuc-P2A-GAL4FF-rGpA-TPS-UAS(8)-EGFP-P2A-Puro^{R}-SV40pA	Fig. 1A, S1 GAL4-UAS, **frameC**-P2A
pLenti-Tet3G-IRES-Neo^{R}	Fig. 2A
pLenti-Tet3G-IRES-Hygro^{R}	Fig. 2A
pMXd3-TRE3G-*c-MYC*-IRES-hmKeimaRed	Fig. 2A
pMXs-*HNF1B*-IRES-Puro^{R}	Fig. 3C
pMXs-*c-MYC*	Fig. 3C

All vectors that we produced and predicted full sequences are available upon request. Numbers in parentheses are the numbers of repeats.

Table S2. Conditions used for splinkerette PCR

Terminal end	Round of nested PCR	Polymerase	Primers (5′ to 3′ direction)	PCR reactions: Temp. & Time	PCR reactions: Cycle
5′TR	1st	Q5 polymerase (NEB) with GC enhancer	CGAAGAGTAACCGTTGCTAGGAGAGACC	98°C, 75 s	1
			CAAAATCAGTGACACTTACCGCATTGACAA	98°C, 20 s<br>64°C, 15 s	2
				98°C, 20 s<br>68°C, 15 s<br>72°C, 2 min	30
				72°C, 7 min	1
	2nd	Q5 polymerase with or occasionally without GC enhancer	GTGGCTGAATGAGACTGGTGTCGAC	98°C, 75 s	1
			CTTACCGCATTGACAAGCACGCCTCACGGG	98°C, 20 s<br>68°C, 15 s<br>72°C, 2 min	30
				72°C, 7 min	1
3′TR	1st	Q5 polymerase without GC enhancer	CGAAGAGTAACCGTTGCTAGGAGAGACC	98°C, 75 s	1
			TAAATAAACCTCGATATACAGACCGATAAA	98°C, 20 s<br>64°C, 15 s	2
				98°C, 20 s<br>68°C, 15 s<br>72°C, 2 min	30
				72°C, 7 min	1
		or KOD Fx (TOYOBO)	CGAAGAGTAACCGTTGCTAGGAGAGACC	94°C, 2 min	1
			TAAATAAACCTCGATATACAGACCGATAAA	98°C, 10 s<br>68°C, 1 min	30
	2nd	Q5 polymerase without GC enhancer	GTGGCTGAATGAGACTGGTGTCGAC	98°C, 75 s	1
			ATATACAGACCGATAAAACACATGCGTCAA	98°C, 20 s<br>68°C, 15 s<br>72°C, 2 min	30
				72°C, 7 min	1
		or KOD Fx	GTGGCTGAATGAGACTGGTGTCGAC	94°C, 2 min	1
			CAGACCGATAAAACACATGCGTCAA	98°C, 10 s<br>68°C, 1 min	30

Table S3. Primers for real-time PCR

Human *OSBPL9* forward	GCGTCCATCTTCCCTACCAG
Human *OSBPL9* reverse	ACGTGGCTTGGAGAAGTGAG
Human *HPRT1* forward	TGACACTGGCAAAACAATGCA
Human *HPRT1* reverse	GGTCCTTTTCACCAGCAAGCT

References

1. Yusa, K., Zhou, L., Li, M.A., Bradley, A. and Craig, N.L. (2011) A hyperactive piggyBac transposase for mammalian applications. *Proc. Natl. Acad. Sci. U.S.A.*, **108**, 1531-1536.
